# A content analysis of e-cigarette related calls to the Shanghai health hotline, for the period 2014–2019

**DOI:** 10.18332/tid/132594

**Published:** 2021-02-24

**Authors:** Jingwen Dong, Jianshu Dong, Yinghuan Zhang, Zian He, Lili Shi, Yuyang Cai

**Affiliations:** 1 School of Public Health, Shanghai Jiao Tong University School of Medicine, Shanghai, China; 2 Shanghai Municipal Center for Health Promotion, Shanghai, China; 3 Shanghai Jiading District Center for Disease Control and Prevention, Shanghai, China; 4 Xinhua Hospital, Shanghai Jiao Tong University School of Medicine, Shanghai, China; 5 China Institute for Urban Governance, Shanghai Jiaotong University, Shanghai, China

**Keywords:** electronic cigarettes, Shanghai health hotline, tobacco legislation, public concerns, e-cigarettes

## Abstract

**INTRODUCTION:**

The health hotline (12320) of Shanghai, not only offers residents information about health knowledge, policies and regulations, but also serves as a channel for public supervision on health issues. This study explored the content of calls towards the Shanghai health hotline (SHH) related to e-cigarettes.

**METHODS:**

The call sheets related to e-cigarette received by SHH were collected from 2014 to 2019. Voice conversations were recorded by the management system of SHH and the telephone operators then converted the recordings into text to collect the information of residents. We used a natural language processor, ROST-CM6.0 to clean up and create words dictionaries and analyzed the text using a text-mining method to identify themes and other useful details.

**RESULTS:**

Among the 491 call sheets, 87.4% were for consultation and 7.5% for complaint. The issue that Shanghai citizens were concerned about most was ‘whether the e-cigarette belongs to the jurisdictional scope of *the Amendment*’, and 76.6% of the call sheets were related to this particular concern. Other concerns were ‘whether e-cigarettes are harmful or not’ (9.4%), ‘can e-cigarettes help people quit smoking or have side effects’ (6.1%), ‘whether e-cigarettes can be sold openly in shopping malls and where can we buy e-cigarettes’ (2.2%) and ‘can minors buy e-cigarettes’ (1.0%).

**CONCLUSIONS:**

The number of call sheets about e-cigarettes received by SHH has seen a significant increase since *the Amendment* was implemented with questions primarily focused on ‘if electronic cigarettes belong to the scope of tobacco control’.

## INTRODUCTION

China manufactures 80% of the world’s e-cigarettes^1^, while it is also the largest consumer of tobacco^2^. Strong e-cigarette production capacity and huge potential market demand have witnessed the rapid development of the e-cigarette market in China. It is predicted that the e-cigarette market in China will exceed 9 billion RMB (Chinese Renminbi) in 2021^3^.

Accordingly, there has been a rise in the use of e-cigarettes by Chinese people. The 2018 China Adult Tobacco Survey showed that 48.5% of respondents (aged ≥15 years) had heard of e-cigarettes, 5.0% had used e-cigarettes, and the current usage rate of e-cigarettes was 0.9%^4^. In 2015, the corresponding percentage was 40.5%, 3.1% and 0.5%, respectively^4^.

The SHH not only offers residents information about health knowledge, policies and regulations, but also serves as a channel for public supervision on health issues. Established in 2006, the SHH has handled consultations, help-seeking, suggestions, and complaints about illegal smoking in Shanghai.

This article analyzed the call sheets related to e-cigarettes in the SHH from 2014 to 2019, concluded the quantitative change of call sheets, and identified areas of concern.

## METHODS

### Data collection

The service management department of the SHH provided the overall systemic support for the daily work flow. Voice conversations were recorded by the management system of the SHH and the telephone operators then converted the recordings into text to collect the information of residents’ appeals. All text documents were noted as a call sheet. The key term of ‘e-cigarette’ was used to search the related call sheets in the database.

We collected 491 call sheets related to e-cigarettes over the past five years (30 June 2014 to 30 June 2019). Each call sheet included four items: 1) Identification – the time and sequence number that were generated for every call sheet, e.g. ‘20170301005078’. This number was used to identify and record the specific time and date of the call; 2) Type – the contents of the calls were divided into five categories – complaint, help-seeking, consultation, suggestion, and others; 3) Descriptive information – this item contained a context-specific description of the calls, such as the details of the reported problems, the appeals of the caller and the replies from the call center; and 4) Feedback – it recorded the subsequent processing results from relevant government departments.

### Context analysis

To make better use of the information in call sheets, we adopted the text-mining technology, a frequently-used method for context analysis on social media, news reports and in literature. We used a natural language processor, ROST-CM6.0, to clean up and create word dictionaries. Meanwhile, topic modeling and clustering were applied in the study to extract the main ideas of the text and facilitate analysis.

The number of call sheets regarding e-cigarettes received by the SHH for each month in the observational time period, as well as the classification of the call sheets, were characterized using descriptive statistics. Mann-Whitney U test assessed the significance of changes in the number of call sheets about e-cigarettes before and after 1 March 2017; analysis was conducted using SPSS 22.0.

## RESULTS

**Figure 1:**
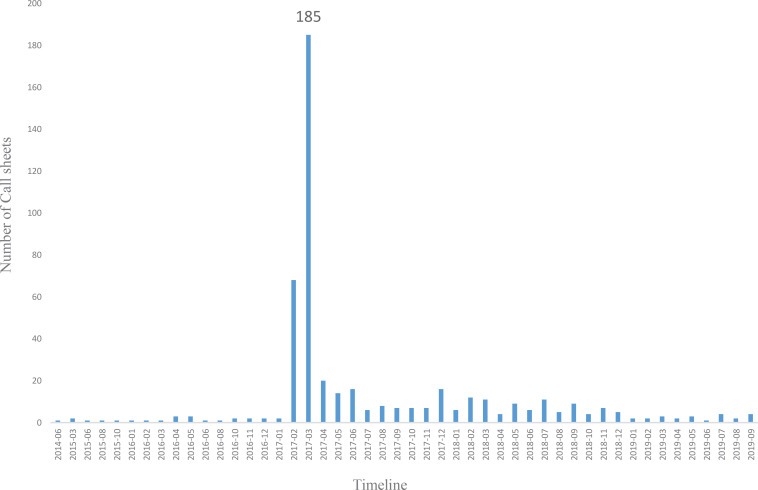
Changes in the number of call sheets of e-cigarette from 30 June 2014 to 30 June 2019 in the Shanghai health hotline

During the observational time period, the SHH received a total of 491 call sheets related to e-cigarettes, 8.2 cases monthly. The number of call sheets increased to 44 on 1 March 2017 (Figure 1). Taking the peak date as a boundary, the average monthly acceptance of sheets related to e-cigarette before the boundary was 0.4, and after the boundary, the number was 15.3, presenting a significant growth (p<0.01).

Among all the 491 call sheets, 429 (87.4%) were for consultation, 37 (7.5%) for complaint, 13 (2.6%) for suggestion, 10 for help-seeking (2%) and 2 for other (0.4%). In March 2017, consultation accounted for 94.6% of the total 185 call sheets with the highest concentration of call sheets feedback.

Analyzing word frequency and semantics of specific content of the call sheets, e-cigarette (632 times), tobacco control (252 times), scope (164 times), regulations (161 times), indoor (132 times) and public places (115 times) were mentioned frequently. The issue that Shanghai citizens were concerned about most was ‘whether the e-cigarette belongs to the jurisdictional scope of *the Amendment*’ (76.6%). Other concerns were ‘whether e-cigarettes are harmful or not’(9.4%), ‘can e-cigarettes help people quit smoking or have side effects’ (6.1%), ‘whether e-cigarettes can be sold openly in shopping malls and where can we buy e-cigarettes’ (2.2%) and ‘can minors buy e-cigarettes’ (1.0%).

## DISCUSSION

Smoking is a major public health problem that China faces. Comprehensive smoke-free legislation is the only effective way to prevent the hazard of environmental tobacco smoke^5^. In 2010, Shanghai took the lead in enacting the Regulations of Shanghai Municipality on Smoking Control in Public Places^6^. According to a survey in 2013, Shanghai citizens expressed high level of support for tobacco control policies^7^. To further the previous ‘Smokeless under the roof’ legislation, *the Amendment* of the regulations (referred to as ‘*the Amendment*’) was formalized on 1 March 2017, which prohibited smoking in indoor public places, indoor workplaces and public transportations^6^. As *the Amendment* was promoted, the number of e-cigarette-related call sheets received by the SHH increased significantly and reached a peak at the effective date. This increase reflected the public’s concern about the applicability of e-cigarettes within the context of *the Amendment* as identified by the content analysis of the call sheets.

In October 2017, a national standard for electronic cigarettes was set up as a project by National Standards Committee of China. The program identified that the State Tobacco Monopoly Administration (STMA) is in charge of e-cigarettes, which means that the government has chosen to regulate e-cigarettes in the range of tobacco products^8^. However, seven months before that, on 1 March 2017, *the Amendment* had already been formally implemented, so e-cigarettes were not explicitly stipulated in *the Amendment*^6^.

Compared with the issue of whether e-cigarettes are in compliance with *the Amendment*, the citizens are less concerned about the potential harm or side effects of e-cigarettes. Previous studies have found that marketing of e-cigarettes, as safer alternatives to cigarettes or cessation aids, is associated with increased e-cigarette usage among young adults^9^. According to a study of teenagers, adolescent e-cigarette users are experiencing symptoms of dependence specific to e-cigarettes^10^. In response to this topic, the Chinese government also formulated regulations to ban the sales of e-cigarettes to minors^11^, especially through the internet^12^. However, the SHH has received very few related appeals (1.0%) on this topic.

## CONCLUSIONS

The number of call sheets about e-cigarettes received by the SHH has seen a significant increase since *the Amendment* was implemented with questions primarily focused on ‘if electronic cigarettes belong to the scope of tobacco control’ and ‘the harms and functions of electronic cigarettes’. Further assessment and follow-up are needed.
